# Rice Genotypes with *SUB1* QTL Differ in Submergence Tolerance, Elongation Ability during Submergence and Re-generation Growth at Re-emergence

**DOI:** 10.1007/s12284-011-9065-z

**Published:** 2011-11-18

**Authors:** Ramani Kumar Sarkar, Bijoya Bhattacharjee

**Affiliations:** Central Rice Research Institute, Cuttack, 753 006 India

**Keywords:** Elongation, Germplasm, Re-generation growth, Rice, *Submergence 1* (*SUB1*), Water stagnation

## Abstract

Submergence tolerance is an important trait where short term flash flooding damages rice. Tolerant landraces that withstand submergence for 1–2 weeks were identified. Due to the heterogeneity in flood-prone ecosystem many different types of traditional rice cultivars are being grown by the farmers. The local landraces adapted to extremes in water availability could be the sources of genetic variation are to be used to improve the adaptability of rice to excess water stress. Greater genotypic variability was observed for plant height, elongation and survival %, absolute growth rate, non-structural carbohydrate retention capacity, chlorophyll content, different chlorophyll fluorescence parameters (FPs) characteristics, and re-generation growth at re-emergence. Twenty days submergence caused greater damage even in *Submergence 1* (*SUB1)* introgressed cultivars compared to the 14 days of submergence. The FPs, carbohydrate content and dry weight at the end of submergence showed positive and highly significant association with re-generation growth. The presence of *SUB1* associated primers, either SC3 or ART5, was noticed even in greater elongating types of rice genotypes. These genotypes possess one or more of the adaptive traits required for the flood-prone ecosystem, which range from temporary submergence of 1–2 weeks to long period of stagnant water tolerance.

**Electronic supplementary material:**

The online version of this article (doi:10.1007/s12284-011-9065-z) contains supplementary material, which is available to authorized users.

## Introduction

Rice is often the only cereal that can be grown in flood prone ecosystem. Uncertainty of rainfall is a major factor affecting the rice yield in India, Bangladesh, and Myanmar with flash flood affecting the plant stand seriously depending on duration of submergence stress which is considered as the third most important constraint to high yield in India, particularly is in the eastern Indian States (Sarkar et al. [2006]; Sarkar et al. [2009a]). Excessive flooding poses risks to human life and is a major contributor to the poverty and vulnerability of marginalized communities especially women and children in poor families (Douglas [2009]). It is estimated that the flood-affected area has more than doubled in size from about 5% (19 million hectares) to about 12% (40 million hectares) of India’s geographic area (World Bank Report [2008]). Adding to these already high risk areas, the climate projections suggest that temperatures, precipitation and flooding, and sea level rise are likely to increase, with adverse impacts on crop yield and farm income in Southeast Asia (Unnikrishnan et al. [2006]; Wassmann et al. [2009]; [INCCA 2010]). Rice in these areas is the major crop providing food for millions of subsistence farming families. Present and anticipated global food demands further necessitate a significant increase in crop productivity on less favorable farmlands and under the adversary of climate change.

Quiescence and elongation are two opposite strategies by which rice adapts to flood depending upon the nature of flooding (Luo et al. [2011]). The ethylene response factors genes *Snorkel1* (*SK1*) and *Snorkel2* (*SK2*) allow rice to adapt to deep water whereas *Submergence1A-1* (*Sub1A-1*) allows rice to acclimatize under flash flooding (Xu et al., [2006]; Hattori et al., [2009]; Nagai et al., [2010]). Both *SKs* genes and *Sub1A-1* are connected with gibberellin biosynthesis or signal transduction, yet deepwater and submergence-tolerant rice seem to have opposite flooding response; namely, escape by elongation or remain stunted under water until flood recedes (Xu et al. [2006]; Hattori et al. [2009]; Sarkar and Panda [2009]; Bailey-Serres et al. [2010]; Bailey-Serres and Voesenek, [2010]). Introgression of *SUB1* QTL into ‘Swarna’ greatly enhanced its survival under submergence, and plant productivity under flash flood conditions (Neeraja et al. [2007]; Sarkar et al. [2009b]). Our wide-ranging on farm and on station trials showed that under normal conditions both cultivars have similar grain yield potential whereas under complete submergence (submergence period varied between 3 and 14 days in different locations), a yield advantage of 1.65 t / ha (an average of 0.81 t / ha over five locations) were obtained from Swarna-Sub1 compared to Swarna (Sarkar et al. [2009b]). Subsequently several submergence tolerant mega varieties namely IR64-Sub1, SambaMahsuri-Sub1, Thadokkam1-Sub1 and BR11-Sub1 were developed (Singh et al. [2009]; Iftekharuddaula et al. [2011]). Swarna-Sub1 was released in India, Indonesia and Bangladesh; BR11-Sub1 was released in Bangladesh; and IR64-Sub1 was released in the Philippines and Indonesia. Breeders are now using the *SUB1* locus to develop tolerant rice varieties for submergence-prone areas in Asia and Africa (Singh et al. [2010]).

Rice plants that exhibit only limited elongation during submergence often show tolerance to flash flooding. The ideotype is not suitable if water level increases and then (i) stays at that level (ii) recedes only partly or (iii) recedes but then rises again and continues for longer duration (Sarkar et al. [2006]). Analysis of flooding pattern in rainfed lowland of Southeast Asia reveals that about 20 million ha comes under medium-deep to deep and very deep ecology based on water stagnation. Here the ideal response to flooding is submergence tolerance (survival under water) together with some elongating ability (Mackill et al. [2010]; Bailey-Serres and Voesenek [2010]). To identify novel sources of tolerance, we have conducted a germplasm survey with allele-specific markers targeting *SUB1A* and *SUB1C*, two of the three transcription-factor genes within the *SUB1* locus along with some physiological traits.

Due to the heterogeneity in flood-prone ecosystem many different types of traditional rice cultivars are being grown by farmers. These cultivars are low yielder but possess one or more of the adaptive traits required for this ecosystem, which range from temporary submergence of one to two weeks, long periods of stagnant water, or daily tidal fluctuations that may sometimes cause complete submergence as in coastal areas. So tolerance to both submergence and to waterlogging may further increase the rice production in marginal rice growing areas. So far, only one submergence tolerant landrace (FR13A) has been extensively exploited in breeding as well as in mechanistic studies, because of its higher level of tolerance compared with all other genotypes that were tested before. Further, recently it was identified a few more tolerant genotypes distinct from FR13A, in being agronomically more desirable (please see Additional file 1: Figure S1 and Additional file 2: Figure S2). This could probably provide better donors and sources of new genes, as some of these genotypes showed better performance than FR13A. Targets that may aid in this objective include the generation of rice genotypes that combine submergence tolerance with tolerance of other abiotic stresses that match local farmers’ preference. Greater efforts are now being devoted to identify more sources and understand the bases of such tolerance. Cultivars are needed that have faster growth after flooding so that it could produce sufficient biomass in a shorter period. Regeneration capacity of submerged rice seedlings is crucial for higher productivity (Panda et al. [2008a]). In the present investigation an attempt was made to identify rice genotypes that differ in submergence tolerance, elongation capacity and re-generation growth.

## Results

### Testing of rice genotypes for *SUB1*

ART5 a closely linked marker when used a 200 bp fragment of *SUB1* found in the promoter region of *SUB1C* in 11 rice genotypes such as Swarna-Sub1, IR64-Sub1, SambaMahsuri-Sub1, INGR04001, INGR08110, AC258830, AC42088, AC20431-W, INGR08109, INGR08111 and FR13A (Table 1, please see Additional file 1: Figure S1 and Additional file 2: Figure S2). The presence of this primer was absent in all other genotypes used in this investigation. The primer SC3 closely linked with *SUB1A* showed distinct band in 15 genotypes. A few genotypes Swarna-Sub1, IR64-Sub1, Sambamahsuri-Sub1, AC258830, AC42088, INGR08109, INGR08111 and FR13A showed distinct band both for SC3 and ART5. The primer SC3 did not show any marker associated band in INGR08110, INGR04001 and AC20431-W. Two primers such as SC3 and ART5 did not show any markers associated bands in susceptible cultivars IR42 and Swarna.

**Table 1:** Survey of rice germplasm with *SUB1A* and *SUB1C* specific primers SC3 and ART5, respectively and plant height, elongation of shoot and survival percentage due to 14 and 20 days of submergence.

Cultivars	Primers		Plant height(cm)		Elongation(%)		Survival(%)	
	SC3	ART5	14 DS	20 DS	14 DS	20 DS	14 DS	20 DS
Swarna-Sub1	(+)	(+)	37 ± 0.8	39 ± 1.3	54 ± 7.4	64 ± 2.5	88 ± 9.0	12 ± 2.5
IR64-Sub1	(+)	(+)	42 ± 4.0	50 ± 3.7	52 ± 11.8	62 ± 1.7	90 ± 3.3	30 ± 8.5
SambaMahsuri-Sub1	(+)	(+)	45 ± 4.1	48 ± 3.0	57 ± 10.6	69 ± 12.1	66 ± 7.8	14 ± 5.0
INGR04001	(–)	(+)	63 ± 3.7	69 ± 3.0	81 ± 18.0	86 ± 5.4	90 ± 3.9	76 ± 5.0
INGR08110	(–)	(+)	63 ± 3.9	71 ± 2.4	89 ± 17.2	101 ± 15.6	87 ± 6.3	68 ± 3.0
AC38575	(+)	(–)	56 ± 1.4	70 ± 3.7	73± 7.5	88 ± 14.8	90 ± 7.1	84 ± 4.5
AC37887	(+)	(–)	53 ± 3.8	68 ± 2.6	88 ± 13.0	91 ± 17.2	88 ± 7.7	84 ± 6.5
IC258990	(+)	(–)	58 ± 4.0	64 ± 2.1	70 ± 11.5	81± 18.0	96 ± 5.3	83 ± 9.0
AC258830	(+)	(+)	60 ± 5.3	66 ± 5.4	74 ± 7.3	97 ± 13.8	92 ± 2.2	88 ± 9.0
AC42087	(+)	(–)	63 ± 2.2	67 ± 2.6	78 ± 7.3	81 ± 9.4	96 ± 5.2	85 ± 6.9
AC42088	(+)	(+)	54 ± 3.0	68 ± 1.3	59 ± 9.0	102 ± 18.9	98 ± 2.3	74 ± 5.7
AC20431-W	(–)	(+)	59 ± 3.2	62 ± 0.8	77 ± 5.9	95 ± 5.2	91 ± 8.4	73 ± 8.5
AC20431-B	(+)	(–)	53 ± 5.7	59 ± 2.4	65 ± 6.7	77 ± 9.1	96 ± 3.9	83 ± 5.5
INGR08113	(+)	(–)	84 ± 1.3	108 ± 5.6	137 ± 15.6	186 ± 10.7	84 ± 9.3	71 ± 4.0
INGR08109	(+)	(+)	96 ± 7.2	113 ± 5.6	147 ± 15.6	163 ± 12.3	90 ± 8.2	77 ± 11
INGR08111	(+)	(+)	85 ± 6.4	107 ± 2.9	157 ± 13.3	194 ± 22.8	81 ± 6.6	53 ± 13
AC42091	(+)	(–)	94 ± 5.3	109 ± 7.3	130 ± 9.0	156 ± 10.7	83 ± 9.3	77 ± 7.0
Swarna (Susceptible check)	(–)	(–)	53 ± 1.6	NB	106 ± 18.6	NB	3 ± 1.5	0
IR42 (Susceptible check)	(–)	(–)	58 ± 3.6	NB	113 ± 12.6	NB	5 ± 1.6	0
FR13A (Tolerant check)	(+)	(+)	56 ± 4.5	59 ± 0.7	61 ± 5.4	63 ± 4.8	93 ± 3.3	78 ± 4.6
**Mean**			**62**	**71**	**89**	**103**	**81**	**60**
LSD*p<0.05			5	6	20	16	10	9

DS, days after submergence; NB, not obtained; Data are presented as mean ± standard deviation based on two years replication wise average data; Numbers of replication, 3. (+), designates presence; (-), designates absent.

### Plant height, elongation and Survival under submergence

Rice genotypes used in this investigation exhibited distinctively variable responses to submergence in terms of visible injury, underwater elongation and plant survival (Table 1). Genotypes INGR08113, INGR08109, INGR08111 and AC42091 showed greater elongation due to the imposition of submergence, and their leaf tips came out above the water within 10 days of submergence (data not shown). The other genotypes however remained under water during the entire period of submergence. Plant height did not increase much in *SUB1* introgressed cultivars, resulted significantly lower elongation compared to other genotypes. Plant height was 39 cm after 20 days of submergence in Swarna-Sub1 whereas the height was 113 cm in a landrace INGR08109. Elongation percentage varied from 62 to 194% after 20 days of submergence. Greater mortality was noticed even after 14 days of submergence in susceptible cultivars. Among the *SUB1* introgressed cultivars survival percentage was only 66% in SambaMahsuri-Sub1 whereas other two cultivars namely, Swarna-Sub1 and IR64-Sub1 showed 88 and 90 percent survival after 14 days of submergence treatment. Survival percentage decreased in almost all the cultivars due to the imposition of 20 days of submergence treatment. Survival percentage was only 12, 14 and 30 percent in *SUB1* introgressed cultivars respectively in Swarna-Sub1, SambaMahsuri-Sub1 and IR64-Sub1. Survival percentage was significantly greater in all the landraces compared to the *SUB1* introgressed cultivars. Among the landraces survival percentage was minimum in INGR08111 (53%). A few genotypes namely, AC38575, AC37887, IC258990, IC258830, AC42087 and AC20431-B showed more than 80 percent survival even after 20 days of submergence. Survival percentage of tolerant check, FR13A was 78%. No significant differences were notice between FR13A and among these landraces.

### Impact of submergence on total above ground dry matter accumulation

**Figure 1:**
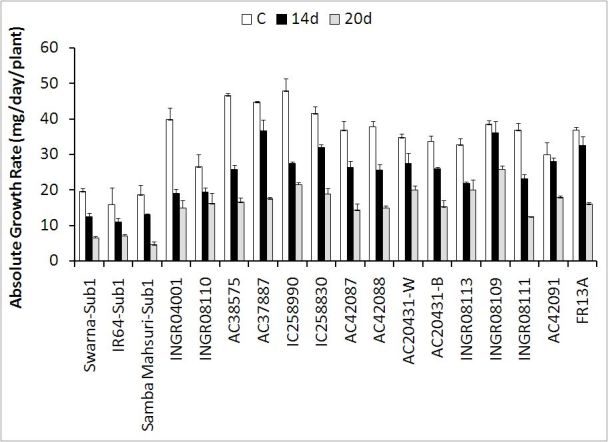
**Absolute growth rate (mg / day / plant) measured from the data taken at the end of submergence of 14 days and 20 days and after 15 days of re-emergence.** To estimate AGR in control plants data were taken simultaneously with 14 days of submergence in non-submerged plots. Bar represents standard deviation. C, non-submerged control; 14 d, 14 days submergence; 20 d, 20 days submergence. Least significant difference at *p < 0.05, Genotype (G) = 2.83; Treatment (T) = 1.10 and G x T = 4.90; Replication, 3

Significant genotypic differences were observed in above ground total dry matter accumulation both under normal and submerged conditions (Figure 1, please also see Table as supplement). In general, total above ground dry matter contents were greater under control condition followed by 14 and 20 days of submergence. The reduction in dry matter content was more than 90 % in susceptible genotypes after 14 days of submergence. The reduction in dry matter contents after 14 days of submergence was 72% in INGR04001 and 78% in AC20431-W whereas in all other genotypes the reduction was between 80 and 88%. Dry matter content decreased drastically due to the imposition of 20 days of submergence mainly to those genotypes, which showed lesser elongation and their leaves tips remained under water for the entire period of submergence. Improvement of dry matter accumulation occurred in INGR08113, INGR08109, INGR08111 and AC42091 at the end of 20 days of submergence compared to the 14 days of submergence as because their leaves tips came out above the water surface. Total dry matter accumulation at 15 days of re-emergence was significantly less in *SUB1* introgressed cultivars compared to the other landraces. Dry matter accumulation of 14 days submerged plants at 15 days of re-emergence was significantly greater in only two rice genotypes INGR08109 and AC37887 compared to the tolerant check FR13A. The scenario changed when compared among the 20 days submerged plants. All the elongating types of genotypes such as INGR08109, AC42091, INGR08113 and INGR08111 showed greater dry matter accumulation compared to the tolerant check FR13A. Among the less elongating types of genotypes two genotypes namely IC258990 and AC20431-W had significantly greater biomass compared to the tolerant check FR13A after 20 days of submergence. Absolute growth rate (AGR, mg / plant) during the period of re-emergence was greater in control plants compared to the 14 days and 20 days of submerged plants (Figure 1). AGR during re-emergence was greater in 14 days submerged plants compared to the 20 days submerged plants. Total dry matter accumulation and AGR were less in *SUB1* introgressed cultivars compared to the other locally grown germplasm lines. AGR was greater compared to FR13A only in few genotypes such as INGR08109, INGR08113, IC258990 and AC20431-W.

### Impact of submergence on total non-structural carbohydrate (NSC) level

**Figure 2:**
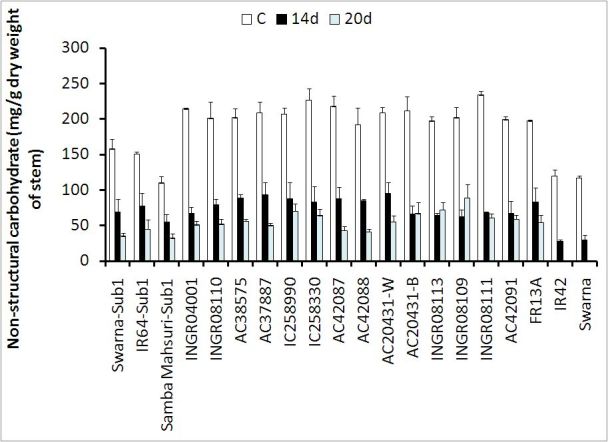
**Non-structural carbohydrate content (mg / g dry weight of stem) was measured at non-submerged control (C) and at the end of 14 days (14 d) and 20 days (20 d) of submergence.** To estimate NSC content in control plants data were taken simultaneously with 14 days of submergence in non-submerged plots. NSC contents did not measure in susceptible cultivars IR42 and Swarna at the end of 20 days of submergence due to the total decomposition of stem. Bar represents standard deviation. C, non-submerged control; 14 d, 14 days submergence; 20 d, 20 days submergence. Least significant difference at *p < 0.05, Genotype (G) = 8.15; Treatment (T) = 3.16 and G x T = 14.12; Replication, 3

In general NSC concentrations before submergence were higher in tolerant genotypes compared to the susceptible genotypes with one exception (Figure 2). The differences of NSC concentrations were non-significant between tolerant SambaMahsuri-Sub1 and susceptible cultivar IR42. Submergence resulted in a remarkable depletion of soluble carbohydrates in the shoots of both tolerant and susceptible cultivars (Figure 2). The level of depletion of carbohydrates was lower in tolerant cultivars compared to the susceptible cultivar, which further widened the differences in carbohydrate levels between tolerant and susceptible cultivars especially after submergence as evident in the % change in carbohydrate contents between control and submerged samples. Reduction of NSC content after 14 days of submergence was 49-56% in *SUB1* introgressed cultivars, 54-69% in other submergence tolerant types, 66-71% in elongating types and 74-77% in susceptible types. Among the non-elongating tolerant types of genotypes the reduction of NSC concentration after 20 days of submergence was low in IC258990 (66%) compared to the tolerant check FR13A (73%) and other tolerant landraces.

### Impact of submergence on pigment content and certain chlorophyll fluorescence parameters

**Figure 3:**
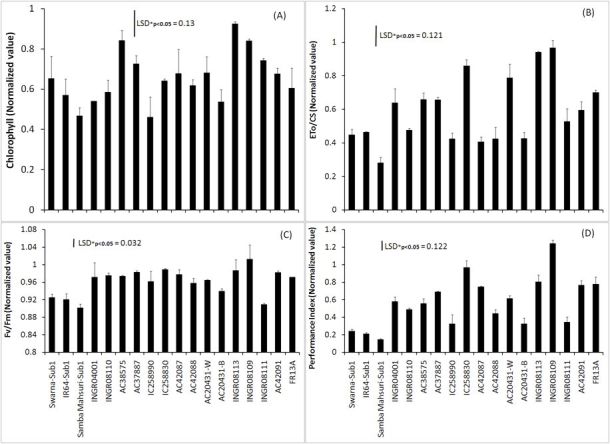
**Normalized values (values after 14 days of submergence/values at corresponding control condition) of chlorophyll content and different chlorophyll fluorescence parameters.** Bar represents standard deviation. LSD, least significance difference. Replication, 3

Chlorophyll level and different chlorophyll fluorescence parameters were studied after 14 days of submergence. Due to the decomposition of leaves of susceptible genotypes data on susceptible cultivars were not obtained. Submergence caused a greater reduction of chlorophyll content (Figure 3**)**. There were great genotypic differences in retention of chlorophyll level among the tolerant rice genotypes. Normalized values of chlorophyll content found greater in elongating types of genotypes namely INGR08113 (0.925), INGR08109 (0.840), and INGR08111 (0.743). Among the less elongating types of genotypes normalized values of chlorophyll content was greater in AC38575 (0.841), followed by AC37887 (0.726). The normalized chlorophyll values ranged between 0.468 and 0.653 among *SUB1* introgressed cultivars. There were great variations even in the values of Fv/Fm ratio between control and submergence among the genotypes (Figure 3). It ranged from 0.901 (e.g. SambaMahsuri-Sub1) to 1.012 (e.g. INGR08109). The normalized values of Fv/Fm ratio were in lower range in *SUB1* introgressed cultivars compared to the other locally grown germplasms except INGR08111. Likely most of the landraces maintained greater values of electron transport capacity per unit cross section (ETo/CS) and overall chloroplast performance index (PI) of Photosystem II (PS II) compared to the *SUB1* introgressed cultivars.

### Correlation studies

The chlorophyll content and different fluorescence parameters (FPs) showed significant association among themselves (Table 2). The chlorophyll content did not show any significant relationship with survival %. On the other hand different FPs especially Fv/Fm and PI showed significant positive association with survival percentage. Highly significant positive association (**P < 0.01) was observed between different FPs and re-generation capacity after submergence. The positive and significant correlations between FPs and dry matter and NSC content after submergence were also observed. Strong positive correlations were observed between NSC contents and survival %. NSC after 14 days of submergence did not show any significant association with other parameters whereas NSC after 20 days of submergence showed significant association with not only survival but also with regeneration growth at re-emergence. The positive and highly significant correlation between dry weight after submergence and regeneration growth at re-emergence suggested that the genotypes maintained greater biomass during submergence could grow faster at re-emergence.

**Table 2:** Correlation coefficients among different physiological parameters with survival and dry matter accumulation

Parameters	Fv/Fm	ETo/CS	PI	NSC14DS	DW14DS	Sur14DS	RDW14DS	NSC20DS	DW20DS	Sur20DS	RDW20DS
Chlorophyll	0.473	0.698	0.586	0.017	0.178	-0.010	0.333	0.431	0.604	0.280	0.571
Fv/Fm	---	0.706	0.890	0.305	0.722	0.431	0.772	0.568	0.427	0.634	0.717
ETo/CS		---	0.826	-0.046	0.425	0.123	0.562	0.674	0.523	0.425	0.689
PI			---	0.099	0.617	0.241	0.771	0.612	0.603	0.544	0.770
NSC14DS				---	0.422	0.602	0.442	-0.123	-0.272	0.491	-0.003
DW14DS					---	0.419	0.761	0.386	0.389	0.760	0.641
Sur14DS						---	0.440	0.242	-0.106	0.591	0.168
RDW14DS							---	0.582	0.510	0.822	0.763
NSC20DS								---	0.676	0.574	0.847
DW20DS									---	0.323	0.886
Sur20DS										---	0.603

The correlation coefficients greater than 0.468 and 0.590 signify the significant association at *p<0.05 and **p<0.01 levels, respectively (degrees of freedom, 16). The correlation study was done excluding the data of susceptible cultivars i.e. Swarna and IR 42 to know whether different physiological parameters either could make distinction among the different tolerant cultivars or not in respect of survival and regeneration growth. Data on chlorophyll, Fv/Fm ratio, ETo/CS and PI were taken after 14 days of submergence along with corresponding control plants. Normalized data of these parameters were used in the correlation studies. In other cases absolute data based on two years average were taken. NSC14DS, non-structural carbohydrate content after 14 days of submergence; DW14DS, total above ground dry matter content after 14 days of submergence; Sur14DS, survival percentage due to 14 days of submergence; RDW14DS, total above ground dry matter content at 15 days of re-emergence affected by 14 days of submergence; NSC20DS, non-structural carbohydrate content after 20 days of submergence; DW20DS, total above ground dry matter content after 20 days of submergence; Sur20DS, survival percentage due to 20 days of submergence; RDW20DS, total above ground dry matter content at 15 days of re-emergence affected by 20 days of submergence.

## Discussion

Rainfed lowlands are subject to numerous types of floods being experienced also in different rice ecologies, ranging from flash flood to medium-deep and deep water; where submergence occurs during early to late vegetative stages for about 1–2 weeks (in flash flood) and 3–6 weeks in medium-deep and deep water conditions. Stagnant flooding occurs in several parts of South-east Asia inundating rice crops to different depths and durations and adversely affecting growth and yield (Ismail et al. [2010]). In areas where typical flash-floods occur (water recedes to lower levels after complete submergence for 1–2 weeks), reduce underwater elongation is beneficial for survival because the elongating plants exhaust their energy reserves and tend to lodge as soon as the water level recedes (Sarkar et al. [1996]; Das et al. [2005]; Sarkar et al. [2009b]; Singh et al. [2009]). However, for most flood-prone areas the ideal ‘ideotype’ is submergence tolerance (survival under water) with partial elongating ability (Sarkar et al. [2006]), particularly for areas where water stagnates in the field following complete submergence. In these areas, flood water tends to stagnate at higher levels (20–60 cm) for longer duration after flash flood, which continues to be the main reason hindering the adaptation of semi-dwarf high yielding cultivars. Cultivars tolerant to various abiotic stresses could ensure the adaptability and hence, contribute considerable in improving the livelihood of the rice farmers in these areas. Cultivars with *SUB1* (e.g. Swarna-Sub1, IR64-Sub1, Samba Mahsuri-Sub1) tolerate complete submergence for over 2 weeks, depending on floodwater conditions (Das et al. [2009]); whereas some other cultivars could withstand 3 weeks of complete submergence with greater variations in plant height and elongation ability under submergence (Table 1). These cultivars maintained greater dry biomass at the end of submergence and re-growth fast during re-emergence (Figure 1). Greater elongation will deliver benefits by restoring contact with the air above the floodwater, thus improving internal aeration for aerobic respiration and allowing for partly aerial photosynthesis. It has also been suggested that shoot elongation may be associated with costs, as energy and carbohydrates are needed for cell division and elongation (Voesenek et al. [2004]; Pierik et al. [2009]). This may ultimately even cause plant death when energy reserves are depleted before reaching the water surface (Das et al. [2005]). In rice culture, therefore, genotypes that slow down growth and respiration when flooded are chosen for cultivation in areas prone to sudden flooding of short duration, whereas genotypes that strongly elongate during flooding are used in flood plains where flooding persists for at least a month (Bailey-Serres and Voesenek [2008]; Chen et al. [2011]; Luo et al. [2011]). Therefore, these new genetic resources tolerant to submergence stress with greater variability of plant height, survival and elongation capacity and greater re-generation growth at re-emergence could help in identifying new genes sources besides *SUB1* and further improve the tolerance level beyond that conferred by *SUB1*.

A quick re-generation growth following submergence is a desirable trait as it ensures production of sufficient biomass for best possible plant productivity (Panda et al. [2008a]). The greater amount of NSC content especially after submergence seemed to have a considerable impact on survival vis-à-vis re-generation of rice plant (Figure 2). The cultivars that maintained a higher carbohydrate content at the time of re-emergence were found to develop greater biomass very quickly (Figure 1). Total nonstructural carbohydrate (sugar + starch) contents after submergence showed highly significant positive association with survival % (Table 2). The maintenance of greater quantities of NSC at the end of submergence depended on the level of NSC contents before submergence and their low consumptive used during submergence (Figure 2). The carbohydrate content of plants was found to be significantly and positively associated with re-generation growth (Panda et al. [2008a]).

Chlorophyll contents decreased due to submergence in rice even in tolerant cultivars (Sarkar et al. [1996]; Sarkar and Panda [2009]). The decline in the values of Fv/Fm ratio reflects a reduction in the ability of PS II to reduce the primary acceptor QA (Panda et al. [2006]). The cultivars tolerant to longer period of submergence maintained the PS II functional and structural integrity better compared to *SUB1* introgression cultivars which showed less survival after 20 days of submergence (Table 1). Therefore, fluorescence characteristics hold the information of survival chance of a plant under long term submergence even within the *SUB1* rice cultivars (Figure 3, Table 2). The plant’s ability to survive for longer duration under extremely high water is, however, related to the storage organs (Crawford [1996]). The cultivars that maintained a higher carbohydrate content at the end of submergence were found to develop new leaves very quickly and accumulated greater biomass during re-emergence (Figure 1).

In most cases, the indel marker ART5 is diagnostic and sufficient for foreground selection (Septiningsih et al. [2009]; Iftekharuddaula et al. [2011]). The *SUB1C* specific allele as FR13A was noticed in eleven tolerant including three *SUB1* introgressed cultivars (Table 1). The *SUB1A* specific allele as FR13A was present in 15 tolerant rice genotypes. Both *SUB1A* and *SUB1C* alleles were present in Swarna-Sub1, IR64-Sub1, SambaMahsuri-Sub1, AC258830, AC42088, INGR08109, INGR08111 and FR13A. Genotypes such as INGR08110, INGR04001 and AC20431-W showed tolerance to complete submergence. *SUB1A* specific primer SC3 did not give any positive result. It showed that *SUB1A* specific primer SC3 alone could not distinguish between tolerant and susceptible genotypes. More *SUB1A* specific primers are to be tested in future to distinguish the genotypes in identifying new genes/alleles (Singh et al. [2010]). Considering the presence of either SC3 or ART5 it appeared that almost all the submergence tolerant cultivars possessed *SUB1* QTL with some allelic specific differences. So far SC3 is one of the closest simple sequence repeat (SSR) markers downstream of *SUB1A* (Iftekharuddaula et al. [2011]). In the *SUB1* region, three similar genes encode the AP2/ERF domain: *SUB1A* *SUB1B* and *SUB1C*. In submergence-intolerant rice cultivar, this locus encodes two ERF genes, *SUB1B* and *SUB1C*. The function of *SUB1B* is not clear. *SUB1* region haplotype determines ethylene- and GA-mediated metabolic and developmental responses to submergence through differential expression of *SUB1A* and *SUB1C* (Fukao et al. [2006, 2009]; Xu et al. [2006]). *SUB1A* diminishes ethylene production and GA responsiveness, causing quiescence of growth under submergence. *SUB1C* on the other hand increases ethylene production and GA responsiveness causes greater elongation of the shoot, greater exhaustion of carbohydrate and poor survival. Earlier reports had suggested that *SUB1A* dominated over *SUB1C* triggered down regulation of *SUB1C* (Xu et al. [2006]; Fukao et al. [2006]). Then it was likely that presence of *SUB1A* restricted shoot elongation. Our results confirmed that it was not always valid (Table 1). Singh et al. ([2010]) reported that *SUB1C* was not directly regulated by *SUB1A*. The greater elongation observed in certain rice cultivars which showed the presence of *SUB1A* might be due the action of other genes product. The ethylene response factors *SK1* and *SK2* allow rice to adapt to deep water whereas *SUB1A-1* allows rice plant to flash flooding. *SK1* and *SK2* are up-regulated by the submergence-induced accumulation of ethylene in internodes, consistent with the essential role of ethylene in GA-stimulated underwater shoot elongation (Hattori et al. [2009]). Till date three QTLs on chromosomes (Chr.) 1, 3 and 12 that regulate the shoot elongation have been identified (Hattori et al. [2007, 2008]). *SUB1* QTL which confers submergence tolerance is found in Chr. 9 (Xu and Mackill [1996]). The greater elongation in INGR08113, INGR08109, INGR08111 and AC42091 compared to the FR13A and other *SUB1* (Table 1) introgressed cultivars suggested that some QTLs associated with deepwater responses might be found in those four genotypes even they possessed *SUB1* QTL (Figure 4). Probably in some rice genotypes grown in rainfed flood prone areas both quiescence and elongation, the two opposite strategies work by which rice adapts to short as well as long term flooding.

For submergence tolerance with greater re-generation capacity we already have the range of genetic diversity required for germplasm improvement; application of easy physiological markers would improve the appropriate selection. Use of physiological traits in evaluating segregating materials is somehow complex and time consuming and in most of the cases require extra facility and manpower. The accurate measurement of total NSC (sugar + starch) within plants is time consuming and likely the bottle neck for why this trait has not been used so far though rapid analysis of carbohydrate may be achieved using Near Infrared Reflectance (NIR). We at CRRI are using hand-held chlorophyll fluorescence meter to predict the vitality of photosynthetic apparatus damages due to submergence. Generally we make a visual survey based on chlorosis of leaves, elongation ability, emergence of leaves tips above the water surface straight forwardly not horizontally and canopy cover based on green leaves formation after 15 to 20 days of re-emergence to estimate re-generation capacity. Straight forward emergence of leaves tips was found to be associated with stiff column as observed in INGR08109 (data not provided). Tolerant cultivars had stiff column and plant became straight at de-submergence. The present study suggests that there is great feasibility to utilize these traits by plant breeders for routine screening works.

## Conclusion

Incidences of flooding have been increased in recent years due to the extreme weather events such as unexpected cyclonic heavy rains and outflows of rivers that have inundated wider areas across many regions in Asia. Rice is the only cereal crop that is well adapted to the conditions of waterlogging or partial flooding or complete submergence. However, phenotypic requirements differ depending upon the situation to withstand the excess water stress. The genotypes identified in this investigation have been provided to National Agricultural Research and Extension Systems’ through All India Coordinated Rice Improvement Programme and Eastern India Rainfed Lowland Shuttle Breeding Network Programme (ICAR-IRRI Collaborative Programme). Important traits for flash flooding are survival of the plant due to complete submergence, reduced under water elongation and greater regeneration capacity at de-submergence. Employment of genotypes such as AC37887, IC258990, IC258830 and AC20431-W in plant breeding programme might further improve the submergence tolerance and plant productivity in rainfed lowland flash flood areas. In different rainfed lowland sites plant experience both complete submergence and waterlogging. In such a condition a perfect blend of elongation and tolerance is required. The genotypes such as INGR08109, INGR08111, INGR08113 and AC42091 could be employed for developing varieties for the locations where both submergence and waterlogging tolerance are required. To identify new QTL/s for submergence tolerance and regeneration capacity development of mapping population is in progress at CRRI using the genotype INGR08109 (vernacular name Atiranga), INGR04001 (vernacular name Khoda) and INGR08110 (vernacular name Kalaputia). The genotypes and physiological traits identified in this investigation could be employed in greater extent to develop high yielding rice cultivars adapted to wide range of rainfed lowland ecosystems.

## Methods

### Plant materials and growth conditions

The experiment was conducted during wet season with twenty rice cultivars at the Central Rice Research Institute (CRRI), Cuttack, India. Cultivars fully tolerant to flash flooding are rare. Only 0.1% of 15,000 rice genotypes tested for submergence tolerance at CRRI survived 12 days of complete submergence (unpublished information). Fourteen genotypes were choosen from the submergence tolerant type based on their elongation ability under submergence and grain charateristics (please see the Additional file 1: Figure S1 and Additional file 2: Figure S2). Among the other six cultivars, three were *SUB1* introgressed high yielding varieties namely Swarna-Sub1, IR64-Sub1 and SambhaMahsuri-Sub1 and three cultivars were used as checks. FR13A was used as tolerant check whereas IR42 and Swarna were used as susceptible checks. The experiment was conducted in alluvial sandy clay loam soil of the Mahanadi River delta (pH 6.7, organic C 0.85%**,** total N 0.01%, avialable P 25 kg/ha, and available K 130 kg/ ha) during the wet seasons of 2009 and 2010. Seeds of the twenty cultivars were sown in a the field tanks (l x b x h : 40 m x 8 m x 0.8 m) @ 4–5 seeds per hill in lines that were 20 cm apart and with 15 cm between hills. The experimental design was a randomized block design with three replications. Chemical fertilizers as basal were added as N:P:K at 20:20:20 kg per ha, respectively. Three separate nearby tanks were used to conduct the experiment. One field tank was used as control where no submergence treatment was provided and plants were grown as usual under normal condition in which rice is cultivated, i.e. with 5–10 cm of stagnant water above the soil surface. Two plant per hill were maintained after 10 days of sowing and necessary weeding operation was also carried out. Twenty-day-old seedlings were completely submerged in other two tanks for 14 days and 20 days, respectively under 80 cm of water so that at least 50 cm depth of water remained above the plant height at initial time of inundation. The characteristics of the floodwater in terms of light availability were measured at 11:30 h (model LI-189, LI-COR, Lincoln, USA) and water temperature and oxygen concentration were determined at 06:30 h and 17:00 h (model Simplair-F-5, Syland Scientific, Heppenheim, Germany). Light intensity at 50 cm water depth or at the vicinity of canopy level ranged from 245 to 300 quantum (μmol /m^2^ /s), whereas it was 2030–2146 quantum (μmol / m^2^ /s) above the water surface. The oxygen concentration at the same water depth was 2.8–4.4 mg / L at 06:30 h and 5.5– 9.5 mg / L at 16:30 h. The floodwater temperatures varied from 26.7–32.5°C throughout the period of the experiment.

### Plant survival due to submergence, plant height, biomass and non-structural carbohydrates

Survival percentage was calculated as {(numbers of survive hills at 15 days of re-emergence) / (the numbers of hills before submergence)*100}. Plant height was taken after 14 and 20 days of submergence and at respective control plot to determine the elongation percentage due to submergence. Extent of elongation of the plant shoot was determined by subtracting plant height of control plants from that after submergence and expressing it as percentage of plant height compared to the non-submerged condition. Aboveground parts were harvested and oven-dried at 65C for 3 days and biomass was determined. Non-structural carbohydrate (NSC) concentrations of stems were determined in control and submerged plants following the procedure of Yoshida et al. ([1976]). NSC contents decreased due to submergence was determined following the formulae as reduction % = {(NSC before submergence – NSC at the end of submergence) / (NSC before submergence)*100}. Absolute growth rate (AGR, mg / day/ plant) was measure using the formulae i.e. AGR = (Dry matter at 15 days of re-emergence - Dry matter at the end of submergence) / 15.

### Chlorophyll a fluorescence and total chlorophyll

Measurements of chlorophyll a fluorescence were made on fully expanded youngest leaves of three different plants in each plot after 14 days of submergence only during the year 2010. The measurement of chlorophyll (Chl) fluorescence was carried out using a Plant Efficiency Analyzer, Handy PEA (Hansatech Instruments Ltd., Norfolk, UK) and data were recorded from 10 μs up to 1 s with a data acquisition of every 10 μs for the first 300 μs, then every 100 μs up to 3 ms and later every 1 ms. The signal resolution was 12 bits (0–4000). For each cultivar, the Chl a fluorescence transients of 9 individual leaves were measured. Leaves were maintained in darkness for 30 min before taking the data on Chl fluorescence. The maximal intensity of the light source, providing an irradiance saturating pulse of 3000 μmol (photons) / m^2^ / s) was used. From the fast O-J-I-P transients, several bio-energetic parameters were derived according to the equations of the JIP-test using the program *BIOLYSER* (R.M. Rodriguez, Bioenergetic Laboratory, University of Geneva). Different chlorophyll fluorescence parameters like minimal fluorescence (Fo), maximal fluorescence (Fm), variable fluorescence (Fv = Fm - Fo), maximal quantum yield of PS II (PSII) photochemistry (Fv/Fm), electron transport per unit cross area of leaves (ETo/CS) and overall performance index (PI) of PSII were calculated using the software. After measuring the Chl fluorescence characteristics, the same leaves were used for the measurement of chlorophyll content, which comprised both chlorophyll a and chlorophyll b. One hundred milligrams of finely chopped fresh leaves were placed in a capped measuring tube containing 25 mL of 80% acetone, and placed inside a refrigerator (4°C) for 48 h. The chlorophyll was measured spectrophotometrically (UV–VIS spectrophotometer, model SL 164, Elico India Ltd., Hyderabad) following Porra ([2002]). Normalized data (values at 14 days of submergence/values of corresponding control plants) on chlorophyll and different chlorophyll fluorescence parameters are presented.

### Genomic DNA extraction

Total genomic DNA was extracted from 20 rice cultivars. Leaves tissues amounting one hundred mg was homogenized with pestle and mortar with liquid nitrogen and grinded leaf powder was transferred to an Eppendorf tube. Four hundred μl of extraction buffer (at 65°C) was added and incubated at 65°C in a water bath for 30 minutes and extracted twice with 700 μl Chloroform and Isoamyl alcohol (24:1). Supernatant was taken in another tube and precipitated the DNA with 2/3 volume of Iso-propanol. Incubated for 30 minutes at −20°C, spun down for 3 minutes with full speed in microcentrifuge at room temperature. Pellet was washed with 70% ethanol thrice. Re-suspended the DNA in 30-50 μl of T.E buffer or H_2_O and kept at −20°C for future use.

### PCR amplification conditions of screening of rice genotypes for the presence of *SUB1* Locus

PCR analysis was carried out using a programmable temperature cycler (Eppendorf). Reaction was carried out using substrate Genomic DNA of different genotypes of rice. The amplification reaction mixture in a 0.5 ml Eppendorf tube consisted of 50 μl reaction mixture containing DNA (1 μg), 200 μM of each dNTPs (Genetix), 1 μM of each forward and reverse primer (Sigma), 1 unit Taq DNA polymerase (Genetix), 2.0 mM MgCl_2_ (Genetix) and 1 X Taq Buffer (Genetix). Two primers namely, SC3 (Forward- GCTAGTGCAGGGTTGACACA; Reverse- CTCTGGCCGTTTCATGGTAT) and ART5 (Forward – CAGGGAAAGAGATGGGTGGA; Reverse – TTGGCCCTAGGTTGTTTCAG) were employed to conduct a germplasm survey with allele-specific markers targeting *SUB1A* and *SUB1C*, respectively. The markers SC3 and ART5 were used to identify recombinants within the *SUB1* gene cluster as in most of the cases; these two markers are diagnostic and sufficient for foreground selection (Neeraja et al. [2007]; Septiningsih et al. [2009]). After denaturing the genomic DNA template at 95°C for 5 min, PCR was performed with 30 cycles of denaturing at 95°C for 45 s, annealing at 65°C for 45 s, extension at 72°C for 60s, and final extension incubation at 72°C for 15 min. PCR amplified products were separated in a 8% agarose gel at 100 V for 2.5 h in 1 x TBE buffer and stained with ethidium bromide. After electrophoresis, the gel was placed on a UV light box and a picture of the fluorescent ethidium bromide-stained amplified PCR product was taken with a camera (gel documentation system).

### Statistical analysis

Differences between the various parameters assessed in this investigation were compared by ANOVA using CROPSTAT (International Rice Research Institute, Manila, Philippines). Means were compared by the least significance difference test ( LSD, *p < 0.05) provided the *F* test was significant. Associations among different traits were examined by simple correlation and regression analysis using the same software.

## Electronic supplementary material

Additional file 1: Figure S1: Germplasm survey with *SUB1A* specific primer, SC3 (JPG 76 KB)

Additional file 2: Figure S2: Germplasm survey with *SUB1C* specific indel market, ART (JPG 78 KB)
