# Supplementary figures and images for: Rice Genotypes with SUB1 QTL Differ in Submergence Tolerance, Elongation Ability during Submergence and Re-generation Growth at Re-emergence
Source: Rice (N Y). 2011 Nov 18;5:7. doi: 10.1007/s12284-011-9065-z (PMC5520825; doi:10.1007/s12284-011-9065-z)

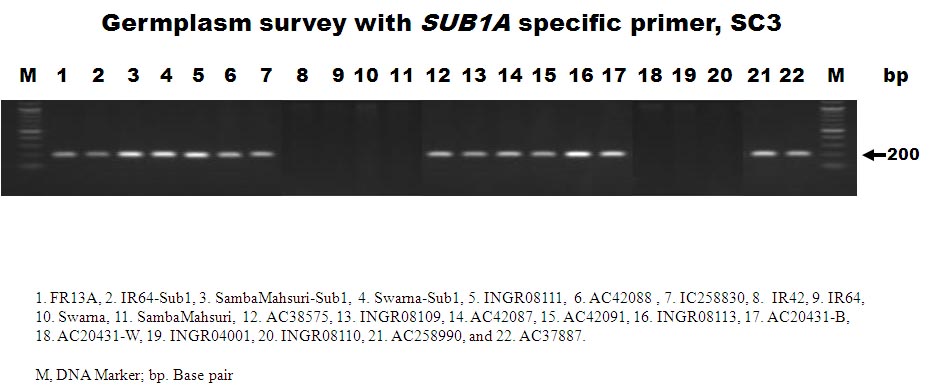

Supplement: Supplementary file 1 — Additional file 1: Figure S1: Germplasm survey with SUB1A specific primer, SC3 (JPG 76 KB) [file 12284_2011_9065_MOESM1_ESM.jpg]

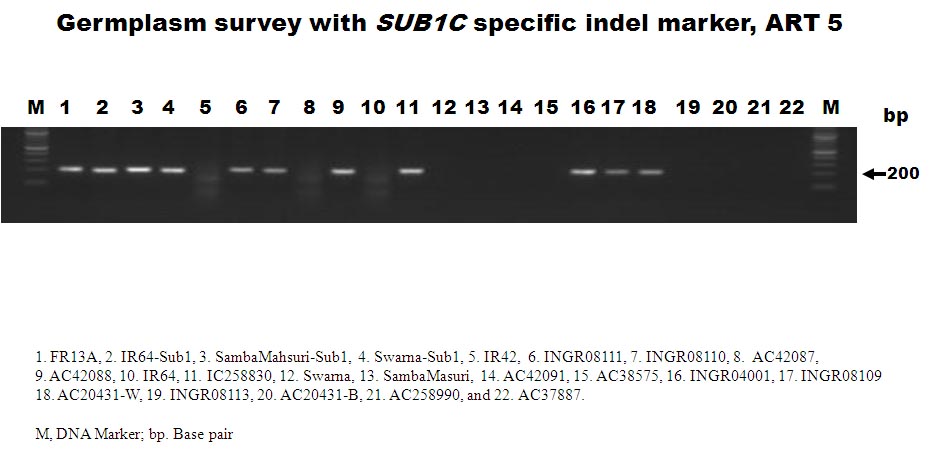

Supplement: Supplementary file 2 — Additional file 2: Figure S2: Germplasm survey with SUB1C specific indel market, ART (JPG 78 KB) [file 12284_2011_9065_MOESM2_ESM.jpg]
